# Association of organizational factors with knowledge of effectiveness indicators and participation in corporate health and productivity management programs

**DOI:** 10.1002/1348-9585.12205

**Published:** 2021-02-11

**Authors:** Hirosuke Takahashi, Masako Nagata, Tomohisa Nagata, Koji Mori

**Affiliations:** ^1^ Department of Occupational Health Practice and Management Institute of Industrial Ecological Sciences University of Occupational and Environmental Health, Japan Kitakyushu Japan

**Keywords:** effectiveness indicators, health and productivity management, health promotion, organization factors, participation

## Abstract

**Objectives:**

The working‐age population is rapidly declining in Japan, so the government has adopted “health and productivity management” (HPM). This policy initiative aims to encourage corporations to view health promotion activities as an investment in their employees’ health. The objective of this study was to examine the association between organizational factors and knowledge of the organization's effectiveness and program participation levels, and to understand the factors that affect effectiveness of corporations’ activities.

**Methods:**

We used data from all corporations that completed the HPM Survey Sheets in 2018 (n = 1800). The explanatory variables were organizational factors: written company‐wide policy, agenda item at management‐level meetings, regular education for managers, and full‐time occupational health staff. The outcome variables were knowledge of the corporation's status on the effectiveness indicators (employees’ exercise habits, risk for high blood pressure, visiting hospital after a health examination, and long‐term sickness absences) and rates of participation in four areas (health education, exercise program, dietary program, and influenza vaccination). The associations between organizational factors and knowledge on effectiveness indicators and rates of program participation were analyzed using multiple logistic regression analysis.

**Results:**

All the organizational factors were related to knowledge of effectiveness indicators, but only some were associated with the program participation indicators in the model, including all explanatory variables.

**Conclusion:**

Enhancing organizational factors may lead to improvement of HPM programs and higher program participation among employees in corporations.

## 1 INTRODUCTION

Japan's working‐age population is rapidly declining because of the decreasing birthrate and aging population.^1^ An extension of the national retirement age is therefore unavoidable to secure a workforce and to maintain social security systems such as medical care and pensions. To make employment possible for older people, it is essential to improve workers’ health and fitness for work.

Well‐designed workplace health promotion programs have a positive return on investment by decreasing sickness absence, reducing medical costs, and increasing job satisfaction.^2^, ^3^ The Japanese government has therefore adopted “health and productivity management” (HPM) as a policy initiative to encourage corporations to view health promotion activities as an investment in the health of their employees, and to help them manage more effectively. The initiative has been led by the Ministry of Economy, Trade and Industry (METI) in collaboration with the Ministry of Health, Labour and Welfare,^4^ and HPM recognition programs are an important element. As of 2020, there are two recognition programs—the HPM Stock Selection program, which selects the most advanced corporation in each industry, and the Certified HPM Corporation Recognition Program, which enables all qualified corporations to apply for recognition. The latter includes both large‐ and small‐ and medium‐sized businesses. The number of corporations participating in both programs is increasing each year.^4^

In the HPM Stock Selection program and under the large corporation sector of the Certified HPM Corporation Recognition Program, corporations are evaluated using self‐administered questionnaires called the HPM Survey Sheets. These are submitted by applicant coporations.^5^ The HPM Survey Sheets are used to evaluate corporations in four areas: “the positioning of HPM in the corporation's philosophy and policies”, “organized frameworks”, “specific systems for implementing HPM”, and “assessment and improvement”.^4^ Additionally, several quantitative indicators associated with HPM performance are assessed. The questionnaire was developed by an expert committee established by the METI, with thorough discussion drawing on the experiences of the committee members. It can therefore be framed as a best practice model for Japanese corporations, but there is no evidence about the extent to which each item is related to actual HPM performance.

Many studies have been conducted on organizational factors influencing the effectiveness of health promotion programs. The importance of factors related to leadership and commitment has been highlighted.^6^, ^7^ The presence of dedicated onsite staff has also been identified as an important factor.^8^ Pronk summarized the similarities and differences in the elements of various health promotion programs outlined in 28 publications. He identified 44 items related to improved outcomes and summarized the elements of a best practice model.^9^ Various surveys and checklists based on many of these findings have been developed and implemented in Europe and the United States,^10^, ^11^, ^12^, ^13^, ^14^, ^15^, ^16^ but little has been done to examine which factors are linked to positive outcomes using real‐world data. One previous study examined the factors in health promotion programs to assess their influence on participation in health assessments and biometric screening, medical costs, and perceptions of organizational and leadership support. It drew on data collected using the scorecard from the Health Enhancement Research Organization (HERO),^10^ a representative checklist used in the United States. The study found that organizational and leadership support was the strongest predictor of success in all examined areas. However, the provision of incentives only predicted increased participation, and program comprehensiveness and program integration were not significant predictors of any of the outcomes.^17^ In another study using data from a competition to find Britain's Healthiest Company, factors such as leadership, incentives, and promotional activities were associated with rate of participation in health promotion programs and employees’ perceptions of program effectiveness.^18^ It is important to carry out a similar check about the factors involved in effective promotion of HPM through the certification programs in Japan.

The METI discloses individual questionnaires from applications to encourage the generation of evidence on HPM. Drawing on the findings of previous studies, the factors that may influence the results of health promotion activities in the workplace can mainly be categorized into two of the four areas measured by the HPM Survey Sheets (Table 1). By analyzing the relationship between the answers to the survey sheet questions and the numerical indicators, it is possible to clarify the organizational factors that affect HPM performance in Japan. These items include indicators of health examination implementation, program participation, lifestyle behaviors, and health status, such as the percentage of employees at risk of high blood pressure, and outcome indicators such as long‐term sickness absenteeism.^5^ Of these, lifestyle behaviors, health status, and the outcome indicators are thought to be strongly influenced by the gender and age structure, but there was insufficient information in our dataset to adjust for these factors. The HPM Survey Sheets ask for input on the status of the corporation as a whole, but it is not always possible for the person in charge of completing the survey to collect all the information requested. In anticipation of such cases, we included indicators for not knowing the overall HPM‐related values for the corporation to assess knowledge of key measures of effectiveness. The importance of continuous evaluation and improvement is emphasized in HPM, and it is likely that significant HPM challenges exist in corporations lacking knowledge of the requested aggregate values.

**TABLE 1:** Organizational factors that affect health promotion program outcomes in two of the four areas of the HPM Survey Sheets

The positioning of HPM in the corporation's philosophy and policies	Commitment of top management
	Leadership of top management
	Integration with the organization's business objectives
Organized frameworks	Utilization of champions at the management level
	Leadership support at the management, middle management, and employee levels
	Employee involvement and establishment of relevant committees
	Dedicated department and staffing
	Ongoing communication that combines many different methods

Abbreviation: HPM, Health and Productivity Management.

In this study, we examined the relationships of key items in the areas of “the positioning of HPM in the corporation's philosophy and policies” and “organized frameworks” in the HPM Survey Sheets with each corporation's knowledge about its status on indicators of effectiveness and program participation. These two areas of the HPM Survey Sheets correspond to the organizational factors influencing the effectiveness of health promotion programs.

## 2 METHODS

### 2.1 Design

This was a cross‐sectional study.

### 2.2 Sample

In the HPM recognition programs, participating corporations voluntarily downloaded the HPM Survey Sheets from the METI’s website, filled in the answers, and submitted them.

We obtained the results from each corporation's response to the 2018 HPM Survey Sheets. The data from all corporations that completed the sheets were used. The details of the composition of the questionnaire have been previously published.^5^

### 2.3 Measures

#### 2.3.1 Explanatory variables

We selected four explanatory variables for analysis. In the area of “the positioning of HPM in the corporation's philosophy and policies”, we used whether there was a “written company‐wide policy for the promotion of HPM” (written company‐wide policy) to measure the organization's commitment. The existence of an “agenda item on the promotion of HPM at management‐level meetings” (agenda item at management‐level meetings) was used to operationalize the integration of HPM initiatives into the organization's business practices. In the “organized frameworks” area, the presence or absence of a “full‐time occupational physician and occupational health nurse” (full‐time occupational health staff) was used to assess the assignment of dedicated departments and staff. The presence or absence of “regular education for managers on health maintenance and promotion measures” (regular education for managers) was used to measure support for middle management as leaders.

#### 2.3.2 Outcome variables

The outcome variables were classified into indicators of knowledge about the corporation's HPM status and indicators of program participation.

In the HPM Survey Sheets, the presence or absence of aggregation of the numerical indicators is first confirmed, and if aggregated, the numerical values are entered. We selected four indicators of knowledge about the status of the corporations, considering the steps and difficulties in identifying each numerical indicator. The first indicator was the percentage of employees engaging in exercise (exercise habits), which is defined in the HPM Survey Sheets as engaging in 30 minutes or more of exercise inducing light sweating on 2 or more days per week. In Japan, corporations have tended to use the standardized questionnaires^19^ set as a part of the specific health examination program based on the Elderly Medical Care Security Act, or questionnaires containing the same items to assess lifestyle habits. Because smoking habits, exercise habits, and adequate sleep are included in the HPM Survey Sheets, and are also included in the standard questionnaire, the difficulty of collecting the information is basically the same for both. Among these, however, smoking habits include unclear treatment of heated cigarettes, and adequate sleep is based on the response that sleep provides adequate rest in the standard questionnaires. These definitions are not clear enough. Furthermore, in the 2018 Survey Sheets, there are no questions regarding programs that address smoking habits or sleep improvement. Therefore, we targeted exercise habits that are clearly defined in the standard questionnaire and for which countermeasure programs are included in the Survey Sheets. The second indicator was the percentage of employees at risk of high blood pressure (at risk of high blood pressure), defined in the HPM Survey Sheets as systolic blood pressure of 180 mmHg or more or diastolic blood pressure of 110 mmHg or more. Indicators in the HPM Survey Sheets that can be ascertained from the analysis of the results of health examinations include percentages of employees who maintain a healthy body weight, percentages of employees at risk of high blood pressure, and percentages of employees at risk of high blood glucose. It is permitted to measure blood glucose at any time in the workplace, but it is difficult to measure fasting blood glucose, so the blood pressure risk rate was selected as most representative. The third indicator was the percentage of employees visiting a hospital for further investigation when their health examination indicated that they should do so (visiting hospital). This indicator cannot be ascertained without following up on employees’ behavior after the health examination. The fourth indicator was percentage of employees with sickness absence or sickness leave because of mental health conditions (long‐term sickness absence). It was defined as absence or leave for longer than 1 month at a time. The information is captured by human resources departments based on medical reports submitted by employees in many corporations. Corporations were assessed as having knowledge of their status on these indicators if they answered “Yes” to the question on the presence or absence of aggregation for each indicator in the HPM Survey Sheets.

In the HPM Survey Sheets, there are five indicators of program participation: the percentage of employees participating in education on health maintenance and promotion (health education), support programs to establish exercise habits (exercise program), and support programs to improve dietary habits (dietary program); the percentage of employees receiving the influenza vaccine (influenza vaccination), and efforts to promote communication. Four of these items were targeted, excluding efforts to promote communication, which tend to be wide ranging and are difficult to interpret. With regard to the content of programs to establish exercise habits and programs to improve dietary habits, the Survey Sheets ask the respondents to answer the programs they provide, to select the most important program among them, and then answer the participation rate for each. To avoid duplicate evaluation of the same program, programs to provide information about a healthy lifestyle, such as group education, are instructed to be excluded from the programs. The indicators of program participation have the following response options: <20%, 20%‐50%, 50%‐80%, ≥80%, and “no information”. The median for all the participation indicators was either in the range of 50% to 80% or ≥80%. We therefore defined a high participation rate as ≥80%. If they answered “no information”, they were regarded as <80%, and therefore not a high participation rate.

#### 2.3.3 Other variables

The activities underlying HPM and health promotion were expected to be influenced by the industry sector and the corporation size. We therefore used information from the questionnaire about industrial classification and number of full‐time employees as adjustment variables. The number of full‐time employees was used as a continuous variable.

### 2.4 Analysis

We used multiple logistic regression analysis to examine the relationships between each of the outcome variables and the explanatory variables. In Model 1, we adjusted only for industrial classification and number of full‐time employees and analyzed the relationship between each explanatory and outcome variable separately. In Model 2, in addition to the adjusted variables in the Model 1, we simultaneously adjusted for all explanatory variables.

For the indicators of knowledge about the corporation's HPM status among the outcome variables, missing values were defined as those for which the presence or absence of aggregation was not mentioned. In the case of indicators of program participation, missing values were defined as those where none of the options including “no information” were selected. Samples with missing values were excluded in the analyses. In addition, we conducted a sensitive analysis by treating the missing value as “No” for Model 2.

We used Stata release 16 (StataCorp LLC, College Station, TX, USA) for all analyses.

### 2.5 Ethical considerations

The METI obtained consent from all responding companies to use these data for research purposes. We also signed a written commitment with the METI to ensure that the data would be kept within the institution and to confirm that we would not disclose the results in a form that made it possible to identify any individual corporation. Additionally, we did not handle individuals’ personal information.

## 3 RESULTS

In total, 1800 corporations (846 listed on the Tokyo Stock Exchange, 10 listed on other exchanges, and 944 not listed on any exchange) submitted the HPM Survey Sheets. At the end of 2019, 3706 corporations were listed in the Japan Exchange Group; 23.1% of these listed corporations submitted the questionnaire (Table 2). There were significant numbers of missing values in the indicators of program participation.

**TABLE 2:** Sample characteristics and descriptive statistics of explanatory and outcome variables

	N	Yes	No	Missing value^a^
Total number of companies completing the HPM Survey Sheets	1800			
Industrial classification				
Wholesale	130			
Retail	203			
Service	615			
Manufacturing and other	852			
Number of full‐time employees				
1‐499	642			
500‐999	343			
1000‐4999	621			
≥5000	194			
Written company‐wide policy^b^		1593	205	2
Agenda item at management‐level meetings^c^		1544	240	16
Full‐time occupational health staff^d^				
No occupational physician or nurse		328	—	
Occupational physician only		10	—	
Occupational nurse only		302	—	
Occupational physician and nurse		1160	—	
Regular education for managers^e^		1333	451	16
Knowledge of the status on the effective indicators^f^				
Exercise habits^g^		1329	418	53
At risk for high blood pressure (≥180/110 mmHg)^h^		1431	318	51
Visiting hospital^i^		947	769	84
Long‐term sickness absences because of mental health problems^j^		1725	39	36
Program participation rate^k^				
Health education		846	664	290
Exercise program		444	1041	315
Influenza vaccination		351	1224	225
Dietary program		412	776	612

Abbreviation: HPM, Health and Productivity Management.

^a^ Missing values: when there is not any answer to the question of whether or not to aggregate.

^b^ Written company‐wide policy: written company‐wide policy for the promotion of HPM.

^c^ Agenda item at management‐level meetings: agenda item on the promotion of HPM at management‐level meetings.

^d^ Full‐time occupational health staff: full‐time occupational physician and occupational health nurse.

^e^ Regular education for managers: regular education for managers on health maintenance and promotion measure.

^f^ Yes: when the answer to "presence/absence of aggregation" is "Yes", No: when the answer is "No".

^g^ Exercise habits: whether the percentage of employees engaging in exercise is reported.

^h^ At risk of high blood pressure (≥180/110 mmHg): whether the percentage of employees at risk of high blood pressure is reported.

^i^ Visiting hospital: whether the percentage of employees visiting hospital to follow up health examinations is reported.

^j^ Long‐term sickness absence because of mental health problems: whether the percentage of employees with long‐term sickness absence because of mental health problems is reported.

^k^ Yes: participation rate ≥ 80%.

### 3.1 Knowledge of status on indicators necessary to understand HPM performance

Table 3 shows the relationships between variables on knowledge of the corporation's status on the selected indicators and the explanatory variables. In Model 1, exercise habits were significantly correlated with all four explanatory variables: written company‐wide policy, agenda item at management‐level meetings, full‐time occupational health staff, and regular education for managers. Knowledge of employees at risk of high blood pressure and knowledge of hospital visits were significantly correlated with all four explanatory variables. Knowledge of long‐term sickness absences was significantly correlated with all explanatory variables except full‐time occupational health staff.

**TABLE 3:** Relationships between the indicators of knowledge about the corporation's HPM status and explanatory variables

	N	Model 1												Model 2											
		Exercise habits^a^			At risk for high blood pressure^b^			Visiting hospital^c^			Long‐term sickness absences^d^			Exercise habits^a^			At risk for high blood pressure^b^			Visiting hospital^c^			Long‐term sickness absences^d^		
		aOR	95% CI	*P* value	aOR	95% CI	*P* value	aOR	95% CI	*P* value	aOR	95% CI	*P* value	aOR	95% CI	*P* value	aOR^f^	95% CI	*P* value	aOR	95% CI	*P* value	aOR	95% CI	*P* value
Written company‐wide policy^e^																									
No	205	reference			reference			reference			reference			reference			reference			reference			reference		
Yes	1593	6.17	4.39, 8.68	<.001	7.63	5.46, 10.66	<.001	3.65	2.56, 5.18	<.001	4.10	2.01, 8.33	<.001	2.67	1.80, 3.96	<.001	2.89	1.95, 4.28	<.001	2.07	1.39, 3.09	<.001	1.60	0.68, 3.77	.28
Agenda item at management‐level meetings^f^																									
No	240	reference			reference			reference			reference			reference			reference			reference			reference		
Yes	1544	5.22	3.79, 7.19	<.001	6.69	4.89, 9.17	<.001	2.83	2.08, 3.85	<.001	4.95	2.57, 9.54	<.001	2.47	1.70, 3.59	<.001	2.92	2.02, 4.22	<.001	1.69	1.19, 2.40	<.05	2.78	1.23, 6.26	<.05
Full‐time occupational health staff^g^																									
No occupational physician or nurse	328	reference			reference			reference			reference			reference			reference			reference			reference		
Occupational physician only	10	3.68	0.44, 30.87	.23	2.56	0.31, 21.18	.38	1.19	0.34, 4.20	.79	0.25	0.03, 2.26	.22	4.96	0.48, 51.65	.18	3.39	0.32, 36.19	.31	1.15	0.31, 4.23	.83	0.16	0.02, 1.71	.13
Occupational nurse only	302	2.26	1.52, 3.35	<.001	2.09	1.36, 3.20	<.05	2.25	1.61, 3.12	<.001	2.73	0.71, 10.52	.15	1.96	1.29, 2.99	<.05	1.71	1.08, 2.72	<.05	1.98	1.41, 2.79	<.001	1.43	0.34, 5.93	.63
Occupational physician and nurse	1160	1.38	1.04, 1.83	<.05	1.43	1.06, 1.92	<.05	1.54	1.19, 1.99	<.05	1.11	0.49, 2.53	.80	1.29	0.95, 1.75	.10	1.30	0.93, 1.82	.12	1.38	1.05, 1.81	<.05	0.67	0.26, 1.69	.39
Regular education for managers^h^																									
No	451	reference			reference			reference			reference			reference			reference			reference			reference		
Yes	1333	3.70	2.87, 4.75	<.001	4.72	3.62, 6.15	<.001	2.46	1.96, 3.10	<.001	4.81	2.45, 9.43	<.001	2.19	1.64, 2.92	<.001	2.52	1.85, 3.43	<.001	1.77	1.37, 2.29	<.001	2.86	1.30, 6.26	<.05

Note

Model 1: adjusted for industrial classification and number of full‐time employees. Model 2: adjusted for industrial classification, number of full‐time employees, and all explanatory variables.

Abbreviation: aOR, adjusted odds ratio; HPM, Health and Productivity Management.

^a^ Exercise habits: whether the percentage of employees engaging in exercise is reported.

^b^ At risk of high blood pressure (≥180/110 mmHg): whether the percentage of employees at risk of high blood pressure is reported.

^c^ Visiting hospital: whether the percentage of employees visiting a hospital to follow up their health examination is reported.

^d^ Long‐term sickness absence because of mental health problems: whether the percentage of employees with long‐term sickness absence because of mental health problems is reported.

^e^ Written company‐wide policy: written company‐wide policy for the promotion of HPM.

^f^ Agenda item at management‐level meetings: agenda item on the promotion of HPM at management‐level meetings.

^g^ Full‐time occupational health staff: full‐time occupational physician and occupational health nurse.

^h^ Regular education for managers: regular education for managers on health maintenance and promotion measures.

In Model 2, all previously significant relationships remained for knowledge about exercise habits (adjusted odds ratio [aOR] 2.67, 95% confidence interval [CI]: 1.80‐3.96 for written company‐wide policy, aOR 2.47, 95% CI: 1.70‐3.59 for agenda item at management‐level meeting, aOR 1.96, 95% CI: 1.29‐2.99 for full‐time occupational health staff [occupational nurse only], and aOR 2.19, 95% CI: 1.64‐2.92 for regular education for managers) and visiting hospital (aOR 2.07, 95% CI: 1.39‐3.09 for written company‐wide policy, aOR 1.69, 95% CI: 1.19‐2.40 for agenda item at management‐level meeting, aOR 1.98, 95% CI: 1.41‐2.79 for full‐time occupational health staff [occupational nurse only], aOR 1.38, 95% CI: 1.05‐1.81 [occupational physician and nurse], and aOR 1.77, 95% CI: 1.37‐2.29 for regular education for managers). Some relationships remained for knowledge of employees at risk of high blood pressure (aOR = 2.89, 95% CI: 1.95‐4.28 for written company‐wide policy, aOR 2.92, 95% CI: 2.02‐4.22 for agenda item at management‐level meeting, aOR 1.71, 95% CI: 1.08‐2.72 for full‐time occupational health staff [occupational nurse only], and aOR 2.52, 95% CI: 1.85‐3.43 for regular education for managers) and long‐term sickness absence (aOR 2.78, 95% CI: 1.23‐6.26 for agenda item at management‐level meeting, aOR 2.86, 95% CI: 1.30‐6.26 for regular education for managers). However, there were no longer significant relationships between knowledge of employees’ status with full‐time occupational health staff and between knowledge of long‐term sickness absences and written company‐wide policy.

### 3.2 Indicators of program participation

Table 4 shows the relationships between the program participation indicators and the explanatory variables. In Model 1, health education participation was significantly correlated with both agenda item at management‐level meetings. Exercise program participation was significantly associated with written company‐wide policy, and dietary program participation with regular education for managers. Influenza vaccination was significantly correlated with agenda item at management‐level meetings and regular education for managers.

**TABLE 4:** Relationships between indicators of program participation and explanatory variables

	N	Model 1												Model 2											
		Health education^a^			Exercise program^b^			Dietary program^c^			Influenza vaccination^d^			Health education^a^			Exercise program^b^			Dietary program^c^			Influenza vaccination^d^		
		aOR	95% CI	*P* value	aOR	95% CI	*P* value	aOR	95% CI	*P* value	aOR	95% CI	*P* value	aOR	95% CI	*P* value	aOR	95% CI	*P* value	aOR	95% CI	*P* value	aOR	95% CI	*P* value
Written company‐wide policy^e^																									
No	205	reference			reference			reference			reference			reference			reference			reference			reference		
Yes	1593	1.56	0.97, 2.50	.07	1.70	1.02, 2.82	<.05	1.34	0.68, 2.65	.40	1.17	0.78, 1.77	.45	0.93	0.54, 1.60	.78	1.57	0.90, 2.75	.11	0.95	0.45, 2.00	.89	0.81	0.50, 1.31	.39
Agenda item at management‐level meetings^f^																									
No	240	reference			reference			reference			reference			reference			reference			reference			reference		
Yes	1544	1.81	1.23, 2.66	<.05	1.12	0.74, 1.71	.59	1.56	0.88, 2.74	.13	1.70	1.12, 2.60	<.05	1.58	1.02, 2.43	<.05	0.91	0.57, 1.44	.68	1.30	0.70, 2.41	.40	1.63	1.01, 2.65	<.05
Full‐time occupational health staff^g^																									
No occupational physician or nurse	328	reference			reference			reference			reference			reference			reference			reference			reference		
Occupational physician only	10	1.65	0.40, 6.75	.49	0.99	0.24, 4.07	.99	0.49	0.05, 4.46	.52	0.57	0.07, 4.69	.60	1.67	0.40, 6.93	.48	0.93	0.23, 3.83	.92	0.48	0.05, 4.39	.51	0.55	0.07, 4.56	.58
Occupational nurse only	302	0.82	0.57, 1.16	.26	0.77	0.53, 1.13	.18	0.98	0.64, 1.49	.91	0.92	0.59, 1.41	.69	0.80	0.56, 1.14	.21	0.74	0.51, 1.08	.12	0.98	0.65, 1.50	.94	0.86	0.56, 1.33	.50
Occupational physician and nurse	1160	1.13	0.84, 1.51	.42	0.80	0.60, 1.08	.15	1.05	0.74, 1.49	.77	1.33	0.96, 1.86	.09	1.09	0.81, 1.47	.56	0.77	0.57, 1.05	.10	1.06	0.75, 1.50	.75	1.27	0.91, 1.78	.16
Regular education for managers^h^																									
No	451	reference			reference			reference			reference			reference			reference			reference			reference		
Yes	1333	1.71	1.30, 2.24	<.001	1.31	0.98, 1.75	.07	1.59	1.11, 2.28	<.05	1.39	1.03, 1.86	<.05	1.58	1.19, 2.10	<.05	1.22	0.88, 1.68	.23	1.53	1.04, 2.24	<.05	1.28	0.92, 1.77	.14

Note

Model 1: adjusted for industrial classification and number of full‐time employees. Model 2: adjusted for industrial classification, number of full‐time employees, and all explanatory variables.

Abbreviation: aOR, adjusted odds ratio.

^a^ Health education: employee health education participation rate ≥80%.

^b^ Exercise program: exercise program participation rate ≥80%.

^c^ Dietary program: nutrition program participation rate ≥80%.

^d^ Influenza vaccination: influenza vaccination rate ≥80%.

^e^ Written company‐wide policy: written company‐wide policy for the promotion of HPM.

^f^ Agenda item at management‐level meetings: agenda item on the promotion of HPM at management‐level meetings.

^g^ Full‐time occupational health staff: full‐time occupational physician and occupational health nurse.

^h^ Regular education for managers: regular education for managers on health maintenance and promotion measures.

In Model 2, the associations of health education with agenda item at management‐level meeting (aOR 1.58, 95% CI: 1.02‐2.43), dietary program with regular education for managers (aOR 1.53, 95% CI: 1.04‐2.24), and influenza vaccination with agenda item at management‐level meeting (aOR 1.63, 95% CI: 1.01‐2.65) remained significant.

### 3.3 Sensitive analysis

When the missing value was analyzed as “No”, the combinations of an explanatory variable and an outcome variable showing a significant difference were added in Model 2. For the effective indicators, there were relationships between written company‐wide policy and long‐term sickness absences. For the program participation indicators, there were relationships between written company‐wide policy and health education, exercise program, and dietary program; between agenda item at management‐level meetings and dietary program, and between regular education for managers and exercise programs.

## 4 DISCUSSION

In this study, using corporations’ responses to the HPM Survey Sheets, we investigated the associations between knowledge of the corporation's status on HPM‐relevant indicators and indicators of program participation with four organizational factors: written company‐wide policy, agenda item at management‐level meetings, full‐time occupational health staff, and regular education for managers. It was considered that these organizational factors corresponded to the organization's commitment, the integration of HPM initiatives into the organization's business practices, the assignment of dedicated departments and staff, and supporting middle management to lead.

We confirmed that each organizational factor was related to knowledge of the corporation's status on the selected indicators. The process of gathering information on indicators related to employees’ health status is essential to understand the needs for programs and the opportunities for improving HPM initiatives. Corporations must be motivated to improve their HPM initiatives to generate effective results, and this motivation is unlikely without management commitment and managerial leadership.^20^, ^21^, ^22^ In corporations that have this motivation, it is necessary to routinely monitor and assess each indicator, which explains why there are associations between organizational factors and the indicators of effectiveness.^23^ All these factors are consistent with the elements of a best practice model suggested by the collective findings of previous studies by Pronk.^9^

The item gauging knowledge of the corporation's long‐term sickness absences was less likely to make a difference than the other outcome variables. This information is usually compiled by human resources departments based on employees’ medical certificates, regardless of whether the corporation promotes HPM. However, it is worth noting that having a written company‐wide policy for the promotion of HPM and HPM as an agenda item at management‐level meetings were associated with knowledge of long‐term sickness absences. To the best of our knowledge, no previous reports have examined the relationships between organizational factors and knowledge about the corporation's status on HPM‐relevant indicators.

We observed that organizational factors were selectively associated with indicators of program participation. Having a written company‐wide policy or full‐time occupational health staff was not associated with any of the program participation indicators. However, HPM as an agenda item at management‐level meetings was associated with health education and influenza vaccination, and regular education of managers was associated with health education and dietary program participation, when all factors were included in one model. Lier et al noted that user rates of health promotion platforms offered in Germany varied greatly (from 0.07% to 100.00%) among client corporations and that organizational support for management to encourage participation in the program increased the program participation rate.^6^ It has also been reported that program participation indicators are associated with support from top management and supervisors,^21^, ^24^, ^25^, ^26^ organizational commitment,^27^ communication with employees,^28^ and a supportive work environment,^29^ including the presence of staff in charge of health promotion departments.

In this study, we found relationships between program participation and organizational factors, which generally supports previous findings. However, previous studies did not consider how each program is associated with different organizational factors. Even if a policy is documented and disseminated, it may not encourage employee participation without an accompanying organizational commitment. The same is true of having full‐time occupational health staff. Integration of HPM initiatives into the organization's business practices is often recognized in best practice models,^30^, ^31^, ^32^ but there have been few specific reports on its effect on program participation rates. We analyzed HPM as an agenda item at management‐level meetings as an indicator of the integration of HPM initiatives into the organization's business practices, and found a significant relationship with two indicators of program participation: health education and influenza vaccination. Health education is often conducted during working hours. It has also been reported that management encouraging vaccination was associated with high rates of vaccination in a worksite‐based program.^26^ These programs may be provided following corporate decision. It is logical to imagine that corporations that regularly provide education on HPM to managers may place more importance on employee health education, but it is unclear why this organizational variable was related to dietary program participation.

A strength of this study is that it used real‐world empirical data from a survey of corporations. This type of study is useful for clarifying the relationships between factors in actual situations. Previous work using surveys and checklists that are used in practice for nonresearch purposes includes studies using data from the Britain's Healthiest Company contest^17^ and the HERO scorecard.^18^ Our study supports these previous studies’ findings that organizational factors were associated with various levels of indicators in real‐world data.

This study had several limitations. First, the HPM Survey Sheets used to provide the study data are self‐administered. The accuracy of the information is therefore uncertain. For example, it is not clear precisely how the respondents aggregated the true data to produce the summary measures requested. Additionally, the purpose of submitting the Survey Sheets is for corporations to be certified in the HPM Stock Selection and the Certified HPM Corporation Recognition Program. There is therefore an incentive to present the organization in a positive light. If there are any major false statements, the certification will be revoked. However, the METI is currently conducting a field survey to introduce actual activities of certified corporations in the HPM Stock Selection as good examples,^33^ and no major false statements in these corporations have been reported to the expert committee. Second, each indicator had many missing values, and these samples were excluded from the analysis in this study. As the results of sensitive analysis, it is possible that the analysis excluding the missing values may have underestimated the relationship between explanatory variables and outcome variables. Third, the respondents were limited to corporations that are implementing or aiming for HPM, and the findings should therefore not be taken to represent typical corporations over a certain size in Japan. Fourth, the research team selected the survey items related to organizational factors identified in previous studies and interpreted the results, but the validity of these items is uncertain. Fifth, the actual questions on program participation in the HPM Survey Sheets first asked for the percentage of employees who have been able to access each program, and then asked for the percentage of those employees who actually participated in the program. In this study, we used the latter question. Since employees in some workplaces may not have had access to the program, our understanding of participation rates in each program may therefore be limited to certain workplaces. Sixth, in this analysis, we used industrial classification and the number of full‐time employees as adjustment factors, but we cannot rule out the possibility that other factors may also have influenced the associations between the indicators.

Despite these limitations, our study suggests that the enhancement of organizational factors, in addition to the provision of health promotion programs, is important for good outcomes from HPM. These findings provide meaningful insights for the future promotion of HPM in corporations and for the effective operation of HPM initiatives by the government.

## 5 CONCLUSION

Using real‐world data, we found that organizational factors affect knowledge of status on indicators of effectiveness and participation rates in health promotion programs in Japan. The impact of each organizational factor varied by indicator and programs. The findings suggest that the enhancement of organizational factors may increase the effectiveness of workplace health promotion initiatives through continuous improvement of programs and high program participation among employees.

## DISCLOSURE

*Approval of the research protocol:* N/A. *Informed consent:* The METI obtained consent from all responding companies to use these data for research purposes. *Registry and the registration no. of the study/trial:* N/A. *Animal studies:* N/A. *Conflict of interest:* The authors have no conflicts of interest directly relevant to the content of this article.

## AUTHOR CONTRIBUTIONS

HT, MN, TN, and KM conceived the study; HT analyzed the data with support from TN and KM. HT and KM led the writing. All authors participated in critically reviewing the study.
